# Endoscopic Obliteration for Bleeding Peptic Ulcer

**DOI:** 10.1155/DTE.4.61

**Published:** 1997

**Authors:** A. W. Włodarczyk, J.J. J. Zawadzki, A.G. G. Gajda, P. Ł. Kamiński, L. Lembas, K. Bielecki

**Affiliations:** 1 State Clinical Hospital Postgraduate Medical Education Center Department of Surgery ul. Czerniakowska 231 Warsaw 00-416 Poland; 2 Państwowy Szpital Kliniczny nr 1 Klinika Chirurgii Ogólnej CMKP ul. Czemiakowska 231 Warszawa 00-416 Poland

## Abstract

A group of 133 patients treated for bleeding peptic ulcer in our Department, is reviewed. Within several hours of admission, all patients underwent upper gastrointestinal tract gastroscopy and obliteration of the bleeding ulcer. Bleeding gastric ulcers were found in 41 patients, and duodenal ulcers in 92 patients. Patients were classified according to the Forrest scale: IA – 11 patients, IB – 49 patients, IIA – 35 patients, lIB – 40 patients. In 126 (94.7%) patients the bleeding was stopped, and 7 required urgent surgery: 3 patients with gastric ulcer underwent gastrectomy, and 4 with duodenal ulcer – truncal vagotomy with pyloroplasty and had the bleeding site underpinned. Fifty-five patients underwent elective surgery: gastrectomy and vagotomy (18 patients with gastric ulcer), highly selective vagotomy (25 patients with duodenal ulcer) and truncal vagotomy and pyloroplasty (12 patients with duodenal ulcer). None of the patients was observed to have recurrent bleeding.


*Diagnostic and Therapeutic Endoscopy*, Vol. 4, pp. 61–64
Reprints available directly from the publisher
Photocopying permitted by license only

© 1997 OPA (Overseas Publishers Association)
Amsterdam B.V. Published under license
in The Netherlands under the Harwood Academic
Publishers imprint, part of The Gordon and
Breach Publishing Group.
Printed in Singapore.

### Endoscopic Obliteration for Bleeding Peptic Ulcer*

A.W. WŁODARCZYK†, J.J. ZAWADZKI, A.G. GAJDA, P.Ł. KAMIŃSKI,

L. LEMBAS and K. BIELECKI

*State Clinical Hospital, Postgraduate Medical Education Center, Department of Surgery, ul. Czerniakowska 231, 00-416 Warsaw, Poland*

*(Received 12 December 1996; Revised 27 January 1997; In final form 26 March 1997)*

**A group of 133 patients treated for bleeding peptic ulcer in our Department, is reviewed. Within several hours of admission, all patients underwent upper gastrointestinal tract gastroscopy and obliteration of the bleeding ulcer. Bleeding gastric ulcers were found in 41 patients, and duodenal ulcers in 92 patients. Patients were classified according to the Forrest scale: IA – 11 patients, IB – 49 patients, IIA – 35 patients, IIB – 40 patients. In 126 (94.7%) patients the bleeding was stopped, and 7 required urgent surgery: 3 patients with gastric ulcer underwent gastrectomy, and 4 with duodenal ulcer – truncal vagotomy with pyloroplasty and had the bleeding site underpinned. Fifty-five patients underwent elective surgery: gastrectomy and vagotomy (18 patients with gastric ulcer), highly selective vagotomy (25 patients with duodenal ulcer) and truncal vagotomy and pyloroplasty (12 patients with duodenal ulcer). None of the patients was observed to have recurrent bleeding.**

*Keywords:* Endoscopic obliteration, Hemorrhage, Peptic ulcer

### INTRODUCTION

Hemorrhage is the most frequent and severe complication of peptic ulcer disease [3,15,16]. Hemorrhage occurs in 25% patients with peptic ulcer at the age of over 50 years, and in 15–29% is the first sign of the disease [2,6].

Mortality in bleeding peptic ulcer is still significant and exceeds 10%, particularly in high risk patients [6,15].

Introduction of endoscopy constituted a great progress in diagnostic and therapeutic management of upper gastrointestinal bleeding. According to some authors, no significantly beneficial influence of urgent endoscopy on prognosis in patients with bleeding peptic ulcer can be proved [5]. Endoscopy is the only available method, allowing for accurate assessment of bleeding site and severity and for selection of further treatment [3,7,15]. In recent years, a variety of endoscopic

* The paper was orally presented at the 15th World Congress of CICD in Seoul, Korea, 1996.

† Corresponding author. Present address: Państwowy Szpital Kliniczny nr 1, Klinika Chirurgii Ogólnej CMKP, ul. Czemiakowska 231, 00-416 Warszawa, Poland. Tel.: (0-48)-(22)-621-71-73.

bleeding management techniques were evaluated [7,8]: mono- and bipolar coagulation, laser photocoagulation, thermal coagulation (*heater probe unit*), and local obliteration.

It was proved that the time of the obliteration influences the effectiveness of treatment [7]. Early endoscopic obliteration allows to stop the hemorrhage in 90% cases and to cancel surgery [15,18].

### MATERIAL AND METHODS

From 2 February 1990 to 31 December 1995, 133 patients, aged 18–83 years (average 50.6 years), were treated in the Department of General Surgery, Postgraduate Medical Education Center, for hemorrhage complicating gastric or duodenal ulcer disease. Bleeding gastric ulcer was found in 41 patients (25 men, 16 women) and bleeding duodenal ulcer was observed in 92 patients (66 men, 26 women). In 2 patients, bleeding gastric and duodenal ulcers were diagnosed.

In patients with signs and symptoms of massive upper gastrointestinal bleeding (60 patients at the time of admission presented the signs of active bleeding, the remaining patients presented the signs and symptoms of recent gastrointestinal bleeding), intensive anti-shock treatment was urgently administered. All patients received 40–80 mg Omeprazole IV and Sucralfate (after endoscopy). All patients underwent gastroscopy within 6 h of admission. This allowed for identifying the site, assessing the severity and attempting obliteration as well as for determining indications for urgent or elective surgery. Patients were assessed according to modified Forrest classification (Fig. 1) [9]. Average amount of blood transfused in patients with Forrest Ia bleeding was 2.1 units and in patients with Forrest Ib – 1.6 units. The remaining patients required no blood transfusion.

From the time of admission, all patients received treatment against Helicobacter pylori (triple therapy: Metronidazole, Amoxicilin, Ranitidine).

Obliteration was achieved by injecting the bleeding site with 20 ml 1 : 10,000 adrenaline solution in 40% glucose in 10–20 injections in or around the ulcer. In the cases when a vessel was identified within the ulcer, 2 ml 1% Ethoxysclerol was injected near the vessel.

In our Department, we have determined the following recommendations as to surgical treatment of bleeding peptic ulcers:

- stomach – resection with truncal vagotomy, and in high risk patients – local excision with truncal vagotomy and pyloroplasty.
- duodenum – local bleeding control (underpinning, excision in the case of ulcers located in the anterior wall of the duodenal bulb), highly selective vagotomy with or without pyloroplasty. Truncal vagotomy is performed in high risk patients. In patients with rapidly healing ulcer, we recommend highly selective vagotomy on elective basis during the same hospital stay.

### RESULTS

In 126 (94.7%) patients treated with endoscopic obliteration hemorrhage was stopped (53 patients with active bleeding and 73 patients with stigmata of recent hemorrhage – SRH). In the group of patients with active bleeding only 7 patients (all Forrest IA) (5.3%) required urgent surgery because of uncontrolled bleeding. In 3 of these patients, local excision with truncal vagotomy and pyloroplasty was performed, and the remaining 4 patients underwent truncal vagotomy and pyloroplasty and underrunning suture was set on duodenal ulcer.

Fifty-five patients underwent elective surgery during the same hospital stay: 18 patients with gastric ulcer had resections with truncal vagotomy performed, and 37 patients with duodenal ulcer underwent either highly selective vagotomy (25 patients) or truncal vagotomy with pyloroplasty (12 patients).

The remaining patients were treated conservatively as: (1) the observed hemorrhage was the first complication of the peptic ulcer disease; (2) these

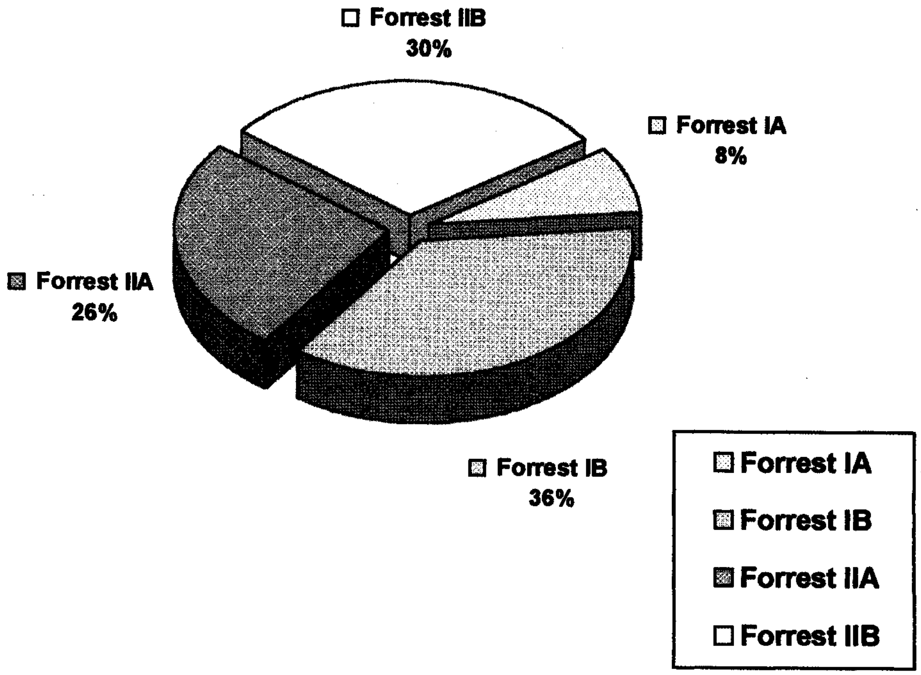

FIGURE 1 Number of patients in respective groups according to modified Forrest classification.

patients had not been treated properly up to the moment of hemorrhage; (3) patients refused to consent for the operation; (4) the underlying risk factors were contraindications for surgery.

None of the patients who were treated conservatively or underwent surgery demonstrated recurrent bleeding. All patients are in follow-up. Time of observation varies from 1 month to 5 years.

In the group of 62 (55 + 7) patients operated for peptic ulcer bleeding, systemic complications occurred in 7 patients (11.3%), and included: myocardial infarction (1 patient; urgent surgery), respiratory complications (6 patients; including 2 patients undergoing urgent surgery).

We observed no complications directly related to the endoscopic obliteration. Mortality rate in patients treated with endoscopic obliteration for upper gastrointestinal bleeding was 2.25%, and in the surgical treatment group – 4.84% (3 deaths in 62 patients: 1 case of massive duodenal bleeding, 1 case of extensive (70%) II° and III° burn, 1 case of multi-organ failure).

### DISCUSSION

Endoscopic treatment of bleeding peptic ulcer is currently the method of choice [15,18]. It is regarded as cost-effective, technically facile, well tolerated and relatively safe [10,15,16,18]. There are reports in the literature of the potential complications of this method: perforation, gastric/duodenal wall necrosis, pancreatic necrosis, induction of new bleeding (not observed in our material), but these occur rather rarely [15,18]. Effectiveness of bleeding management in our Department was 94.7%, which compares well to the data of other authors.

In our patients we observed active ulcer bleeding in 45.1% cases; in the remaining patients, stigmata of recent hemorrhage (SRH) were present. Only 11.7% patients of this group required urgent surgery. We observed no cases of recurrent bleeding after effective obliteration. Asaki *et al.* [1] reported the results of multi-center study performed in Japan that included 332 patients, 90%

of whom had bleeding peptic ulcer. In 52% patients bleeding or SRH were observed, and in 20 cases (6%), bleeding recurred after obliteration. Foster *et al.* reported recurrent bleeding in 42% patients with SRH, half of whom required urgent surgery [4]. Hirao *et al.* reported effectiveness of 93% in the treatment of peptic ulcer bleeding by obliteration with less than 1% patients requiring urgent surgery [11]. Steele *et al.* [13] treated 53 patients with ulcer bleeding with 94% effectiveness rate. Only 11% required another obliteration, and 17% urgent surgery.

We observed some cases of systemic complications (myocardial infarction, respiratory complications, mainly pneumonia). These concerned particularly the patients who had undergone urgent surgery. In our opinion, they were not directly associated with endoscopic procedures. Many authors [8,14,15,18] report cardio-respiratory complications after endoscopy, which can be associated with hypoxia (impaired ventilation during endoscopy).

Overall mortality rate in the group of patients treated for peptic ulcer bleeding was 2.25%, and the one in the surgical treatment group was 4.48%. These are lower than reported in the literature (Woods *et al.* – 10%, Oxner *et al.* – 8.3%, Chen *et al.* – 9%) [12,15,17]. Dobosz *et al.* report mortality rates of 35% and 4.3% in the urgent and elective surgery groups, respectively [3].

We conclude that endoscopic obliteration is a highly effective method in the treatment of bleeding peptic ulcer. In significant number of patients this method allows to change indications for urgent surgery for elective ones. The presented diagnostic and therapeutic management decreases overall mortality and the number of complications associated with bleeding gastric or duodenal ulcers.

### References

[1] Asaki, S., Nishimura, T., Satoh, A., Ohara, S., Shibuya, D., Ogitsu, Y. and Goto, Y. Endoscopic hemostasis of gastrointestinal hemorrhage by local application of absolute alcohol: A clinical study. *Tokohu J. Exp. Med.* 1983; **141**: 373–383.

[2] Bielecki, K. Współczesne poglądy na leczenie chirurgiczne choroby wrzodowej. *Post. Nauk Med.* 1988; **1**: 84–92.

[3] Dobosz, M., Babicki, A., Marczewski, R., Juszkiewicz, P. and Wajda, Z. Wskazania do chirurgicznego leczenia krwawiących wrzodów żołądka i dwunastnicy po endoskopowym zatrzymaniu krwawienia metodą iniekcyjną. Wybrane zagadnienia z chirurgii 1995, wyd. Fundacja – Polski Przegląd Chirurgiczny, Warszawa 1995, str. 53–55.

[4] Foster, D.N., Miloszewski, K.J.A. and Losowsky, M.S. Stigmata of recent hemorrhage in diagnosis and prognosis of upper gastrointestinal bleeding. *Br. Med. J.* 1978; **1**: 1173–1177.

[5] Fromm, D. Endoscopic coagulation for gastrointestinal bleeding. *N. Engl. J. Med.* 1987; **316**: 1652–1654.

[6] Gieroba, J., Czarnecki, J., Szczerbiński, M., Celiński, K. and Danilewicz, W.C. Analiza kliniczna krwawień z górnego odcinka przewodu pokarmowego. *Gastroenterol. Pol.* 1995; **2**: 43–46.

[7] Grzebieniak, Z., Łazarkiewicz, B., Kielan, W. and Woytoń, M. Endoskopowe skojarzone tamowanie krwawień z górnego odcinka przewodu pokarmowego w modyfikacji własnej. Wybrane zagadnienia z chirurgii 1995, wyd. Fundacja – Polski Przegląd Chirurgiczny, Warszawa 1995, str. 50–52.

[8] Hart, R. and Classen, M. Complications of diagnostic gastrointestinal endoscopy. *Endoscopy* 1990; **22**: 229–223.

[9] Heldwein, W., Schreiner, J., Pedrazzoli, J. and Lehnert, P. Is the Forrest classification a useful tool for planning endoscopic therapy of bleeding peptic ulcers? *Endoscopy*, 1989; **21**: 258–262.

[10] Herold, G., Preclik, G. and Stange, F. Gastroduodenal ulcer hemorrhage: endoscopic injection therapy using a fibrin sealant. *Hepato-Gastroenterol.* 1994; **41**: 116–119.

[11] Hirao, M., Kobayashi, T., Masuda, K., Yamaguchi, S., Noda, K., Matsura, K., Naka, H., Kawauchi, H. and Namiki, M. Endoscopic local injection of hypertonic saline-epinephrine solution to arrest hemorrhage from upper gastrointestinal tract. Gastrointest. *Endosc.* 1985; **31**: 313–317.

[12] Oxner, R.G.B., Simmonds, N.J., Gertner, D.J., Nightingale, J.M.D. and Burnham, W.R. Controlled trial of endoscopic injection treatment for bleeding from peptic ulcers with visible vessels. *Lancet* 1992; **339**: 966–968.

[13] Steele, R.J.C., Park, K.G.M., Crofts, T.J. and Li, A.K.C. Adrenaline injection for endoscopic hemostasis in non-variceal upper gastrointestinal hemorrhage. *Br. J. Surg.* 1991; **78**: 477–479.

[14] Steffes, C.P., Sugawa, C., Willson, R.F. and Hayward, S.R. Oxygen saturation monitoring during endoscopy. *Surg. Endosc.* 1990; **4**: 174–178.

[15] Steffes, C.P. and Sugawa, C. Endoscopic management of nonvariceal gastrointestinal bleeding. *World. J. Surg.* 1992; **16**: 1025–1033.

[16] Stocki, W., Baniukiewicz, A., Kamocki, Z., Bandurski, R. and Tołwiński, W. Współczesne metody leczenia w krwawiących owrzodzeniach żołądka i (lub) dwunastnicy. Wybrane zagadnienia z chirurgii 1995, wyd. Fundacja – Polski Przegląd Chirurgiczny, Warszawa 1995, str. 44–47.

[17] Storey, D.W., Bown, S.G., Swain, C.P., Salmon, P.R., Kirkham, J.S. and Northfield, T.C. Endoscopic prediction of recurrent bleeding in peptic ulcers. *N. Eng. J. Med.* 1981; **305**: 915–916.

[18] Sugawa, C. and Joseph, A.L. Endoscopic interventional management of bleeding duodenal and gastric ulcers. *Surg. Clin. Am.* 1992; **72**: 317–334.

